# Sero-prevalence and risk factors for maternal and umbilical cord hepatitis B surface antigenaemia at delivery in a South-West Nigerian State

**DOI:** 10.11604/pamj.2024.49.55.45111

**Published:** 2024-10-25

**Authors:** Babatunde Olaniyi Roiji, Adepeju Dorca Roiji, Olumide Ebeezer Adewara, Musah Yusuf, Olajide Alfred Durojaye, Omotayo Oladele Adeniyi, Micheal Olumide Gbala, Babatola Bakare, Babatunde Ajayi Olofinbiyi

**Affiliations:** 1 Department of Obstetrics and Gynaecology, State Specialist Hospital, Ikole-Ekiti, Ekiti State, Nigeria,; 2 Department of Pharmacology, Federal Teaching Hospital, Ido-Ekiti, Ekiti State, Nigeria,; 3 Department of Obstetrics and Gynaecology, Federal Teaching Hospital, Ido-Ekiti, Ekiti State, Nigeria,; 4 Department of Internal Medicine, Federal Teaching Hospital, Ido-Ekiti, Ekiti State, Nigeria,; 5 Legal Medical Clinic, Legal, Alberta, Canada,; 6 Bayly Family Practice and Walk in Clinic, Bayly Street, Toronto, Canada,; 7 Department of Obstetrics and Gynaecology, University of Medical Sciences Teaching Hospital, Ondo, Ondo State, Nigeria,; 8 Department of Obstetrics and Gynaecology, College of Medicine, Ekiti State University, Ado-Ekiti, Ekiti State, Nigeria

**Keywords:** Sero-prevalence, risk factors, maternal, umbilical cord, hepatitis B antigenemia, delivery, Nigeria

## Abstract

**Introduction:**

hepatitis B is a global public health issue affecting about 2 billion people, with 350 million chronic carriers. There is growing pressure to test pregnant women for hepatitis B in Nigeria, as studies show high rates of the virus in both mothers and children. Objectives: this cross-sectional study aimed at determining the sero-prevalence of hepatitis B surface antigen among pregnant women and their newborns and identifying risk factors associated with hepatitis B surface antigen positivity among pregnant women receiving care in tertiary hospitals in Ekiti State, Southwest Nigeria.

**Methods:**

two hundred women were recruited into the study from Federal Teaching Hospital, Ido-Ekiti, and Ekiti State University Teaching Hospital, Ado-Ekiti between 1^st^ May 2022 and 31^st^ October 2022. The presence of risk factors for hepatitis B infection was sought in the mothers. Maternal venous blood and umbilical cord blood were analyzed for the presence of hepatitis B surface antigen. Data analysis was carried out using a statistical program for social sciences (SPSS) version 28 (SPSS Inc., Chicago, Illinois, USA).

**Results:**

the mean age of the women was 30.43 years (SD 4.524). Thirty-three (16.5%) of the women studied were into hospital-related professions while 6 (3%) were hairdressers. Sixteen (16) of the 200 women tested positive for HBsAg giving a maternal sero-prevalence of 8% while only 1 of the 200 babies tested positive for HBsAg with a neonatal sero-prevalence of 0.5%. The vertical transmission was 6.25%.

**Conclusion:**

high endemicity of hepatitis B virus infection in pregnancy was demonstrated in the study. It has, therefore, become imperative to strengthen the structure of the various preventive arms of hepatitis B infection in pregnancy such as advocacy on increased community awareness and handling of risk factors, widespread vaccination campaigns, and routine testing during prenatal care.

## Introduction

Hepatitis B is a global public health issue affecting about 2 billion people, with 350 million chronic carriers [1,2]. Despite being vaccine-preventable, many developing countries still have a high disease burden [2]. In pregnant women, hepatitis B can be transmitted to the fetus, with a risk of 10% in the first trimester and up to 90% in the third trimester, especially for mothers with HBsAg and HBeAg [3,4]. Nigeria is one of the countries with a high prevalence of hepatitis B, with 18 million infected individuals [5,6]. There's growing pressure to test pregnant women for hepatitis B in Nigeria, as studies show high rates of the virus in both mothers and children [7,8]. However, current tracking systems have gaps. Most studies miss newborns and young infants, making it hard to gauge the true impact of mother-to-child transmission and its effect on national immunization programs [7]. This study sought to determine the sero-prevalence and risk factors for maternal and umbilical cord hepatitis B surface antigenaemia at delivery in a southwest Nigerian State. In a country with high hepatitis B rates, it has become imperative to test both pregnant women and their newborns for the virus. Literature search has also shown a paucity of data on hepatitis B infection in pregnancy in the environment of study. This study revealed the burden of Hepatitis B among pregnant women and the immediate impact of mother-to-child transmission, aiding in developing effective interventions in the area of study.

## Methods

**Study design and setting:** this was a cross-sectional multicentre study conducted at the Departments of Obstetrics and Gynaecology of two major hospitals: the Federal Teaching Hospital, Ido-Ekiti and Ekiti State University Teaching Hospital, Ado-Ekiti. These hospitals serve as referral centres for primary and secondary health care facilities in Ekiti State and neighbouring States in South-western Nigeria. The study was conducted between May 1^st^, 2022 and October 31^st^, 2022.

**Study population:** the study population comprised pregnant women who presented for delivery at the labor wards of the two study centers and met the inclusion criteria. The inclusion criteria were: i) pregnant women with a gestational age of 28 weeks or more (age of viability); ii) those who gave written informed consent to participate; iii) all neonates delivered by the recruited pregnant women.

**Exclusion criteria included:** pregnant women with a gestational age of less than 28 weeks; ii) HIV-positive women; iii) women who did not provide consent for participation; iv) patients on antiviral agents for any indication; v) women with pregnancies complicated by intrauterine fetal death or multiple pregnancies.

**Sample size determination:** the sample size was calculated based on hepatitis B sero-prevalence of 6.0% using the formula by Fisher.


$$N=\frac{z^{2}p\left(q\right)}{d^{2}}$$

Where N is sample size; Z is the standard normal deviation (a constant) which at 95% confidence interval=1.96; p= known prevalence of the disease; q=1-p; d= error margin allowable= 5%;


$$N=\frac{1.96^{2}\times 0.06\left(1-0.06\right)}{0.05^{2}}$$

The sample size was approximated to be 100 patients for each of the study locations. Two hundred consecutive (200) patients were recruited into the study from both study locations between 1^st^ May, 2022 and 31^st^ October, 2022.

**Study procedure:** upon presenting for delivery, a brief history was obtained from each participant, including age, parity, occupation and booking status. Information on specific risk factors for hepatitis B infection, such as previous blood transfusion, history of surgery or dental procedures and past jaundice was also recorded. Participants were screened for hepatitis B surface antigen (HBsAg) using the DiaSpot® rapid screening kit, following the manufacturer´s instructions. The screening test was conducted on maternal blood samples. During the third stage of labour, umbilical cord blood was obtained from the fetal end for HBsAg screening in neonates, following the same procedure used for maternal samples.

**Sample collection and handling:** maternal venous blood samples were collected using a sterile 5 ml disposable hypodermic syringe and needle. After disinfecting the collection site, 5 ml of blood was drawn and transferred into Ethylene Di-amine Tetra-acetic Acid (EDTA) bottles. The samples were centrifuged at 5000 revolutions per minute (rpm) for 5 minutes, and plasma was separated for HBsAg determination. For the neonates, 3 ml of umbilical cord blood was collected using the same sterile technique. The samples were also centrifuged at 5000 rpm for 5 minutes to separate plasma for HBsAg testing. When immediate testing was not possible, plasma samples were stored at 2-8°C and tested within 3 days. DiaSpot® HBsAg test strips (manufactured by DiaSpot Diagnostics, USA) were used to detect HBsAg in plasma samples. For testing, both maternal and fetal samples were allowed to equilibrate to room temperature. Test strips were immersed vertically into the plasma samples for 15 seconds, ensuring not to exceed the maximum line on the strip. Results were read after 15 minutes, with double lines indicating positive samples and a single control line for negative samples.

**Data collection:** data from each participant, including sociodemographic characteristics and relevant clinical history, were collected using a structured proforma. The results of maternal and neonatal HBsAg tests were also recorded. The proforma was administered by the principal investigator or trained research assistants. These research assistants were resident doctors in the Department of Obstetrics and Gynaecology who had received training for the study.

### Definitions

**Dependent variables:** the dependent variables in this study were the hepatitis B surface antigen (HBsAg) status of: i) maternal HBsAg status: this refers to whether the pregnant women tested positive or negative for hepatitis B surface antigen based on the rapid screening test; ii) neonatal HBsAg status: this refers to whether the neonates tested positive or negative for hepatitis B surface antigen, determined from the umbilical cord blood samples. These variables reflect the outcomes being measured to determine the rate of hepatitis B infection transmission from mother to child.

**Independent variables:** the independent variables in the study included factors that could potentially influence maternal and neonatal HBsAg status. These variables are:

**Sociodemographic factors:** i) age of the pregnant women; ii) parity (number of previous pregnancies); iii) occupation (categorized according to the patient´s employment or work status); iv) socioeconomic status (assessed indirectly via occupation and booking status).

**Obstetric and medical history:** i) booking status (whether the patient had antenatal care or not); ii) history of blood transfusion (prior history of receiving blood transfusions); iii) history of surgery or dental procedures (prior surgical interventions or dental treatments); iv) past history of jaundice. These independent variables were considered as potential risk factors for maternal HBsAg positivity and vertical transmission of hepatitis B to neonates.

**Statistical analysis:** all data were entered into a computer and analyzed using SPSS version 28 (SPSS Inc., Chicago, Illinois, USA). Descriptive statistics were used to summarize the data. Cross-tabulations were performed to explore associations between variables, and the chi-square test was used to assess the relationship between categorical variables. Statistical significance was set at p< 0.05, providing a 95% confidence interval.

**Ethical consideration:** ethical approval for this study was obtained from the Ethical Review Board of Ekiti State University Teaching Hospital, Ado-Ekiti. The study adhered to the ethical principles, ensuring the protection of participants' rights and confidentiality. Informed written consent was obtained from all participants prior to their inclusion in the study, and they were fully informed about the study's purpose, procedures and potential risks and benefits.

## Results

**Socio-demographic characteristics of the respondents:** the socio-demographic characteristics of the patients are presented in Table 1. The age of respondents ranged between 16 and 42 years with a mean of 30.43 years (SD= 4.52). The majority of the women (n=149) were between the age range of 25-34 years. Seventy-seven (38.5%) of the respondents were civil servants, 20% were unemployed, 16.5% worked in hospital environments (nurses, health attendants, doctors, and dental technicians), and 3% were hairdressers. The studied women were predominantly Christians (96.5%) of the Yoruba tribe (95%) and the vast majority were married (97%). Ninety-eight percent of the women studied had some form of education; 45.5% were schooled up to tertiary level, while 29.5% and 19.5% attended teachers´ training college/school of nursing and secondary schools respectively. Sixteen (8%) of the studied women tested positive for hepatitis B surface antigen while the remaining 184 women were negative (Table 2). The sero-prevalence of hepatitis B surface antigenaemia was thus 8%.

**Table 1:** socio-demographic characteristics of the respondents

Variable	Frequency N=200	Percent
**Age group (yrs)**		
≤19	5	2.5
20-24	11	5.5
25-29	71	35.5
30-34	78	39.0
≥35	35	17.5
**Occupation**		
Unemployed	40	20.0
Trading	19	9.5
Civil servant	77	38.5
Hospital-related	33	16.5
Hairdressing	6	3.0
Others	25	12.5
**Religion**		
Christianity	193	96.5
Islam	7	3.5
**Tribe**		
Yoruba	190	95.0
Hausa	2	1.0
Others	8	4.0
**Marital status**		
Single	6	3.0
Married	194	97.0
**Marriage setting**		
Monogamous	184	92.0
Polygamous	10	5.0
Single	6	3.0
**Educational status**		
None	4	2.0
Primary	7	3.5
Secondary	39	19.5
Tertiary	59	29.5
Postgraduate	91	45.5
**Total**	200	100%

**Table 2:** vertical transmission of hepatitis B surface antigenaemia

Variable	Frequency	Vertical transmission rate**
HBsAg* Positive mothers	16	6.25%
HBsAg* Positive babies	1	
HBsAg*: Hepatitis B surface antigen		
HBsAg*: Hepatitis B Surface Antigen, **Vertical transmission rate; No HBsAg positive babies/No HBsAg positive mothers x 100		

**Vertical transmission of hepatitis B surface antigenaemia:** only one (0.5%) of the babies in this study tested positive for hepatitis B surface antigen. The remaining 199 babies (99.5%) tested negative. This put the neonatal sero-prevalence of hepatitis B surface antigenaemia at 0.5% and the vertical transmission rate for this study was 6.25% (Table 2).

**The risk factors for hepatitis B surface antigenaemia:** the risk factors for hepatitis B surface antigenaemia are presented in Table 3. The women´s occupation was a significant risk factor for hepatitis B surface antigenaemia. Women who were doing hospital-related jobs and hairdressers were more likely to be surface antigen-positive with the occupation-based prevalence of 18.2% and 16.7% respectively (Table 3, Table 4). There was zero prevalence among unemployed women and traders.

**Table 3:** risk factors for hepatitis B surface antigenaemia

Variable	Maternal HBsAg		Chi-square	Df	P-value
	Positive	Negative			
**Occupation**			11.532	5	0.042
Unemployed	0	40			
Trading	0	19			
Civil servant	8	69			
Hospital-related	6	27			
Hairdressing	1	5			
Others	1	24			
**Scarification/tribal marks/tattoo**			39.652	1	0.001
Yes	5	2			
No	11	182			
**Previous blood transfusion**			0.725	1	1.000
Yes	0	8			
No	16	176			
**Previous surgical/dental procedure**			2.065	1	0.151
Yes	6	40			
No	10	144			
**Past history of jaundice**					
Yes	0	0	-	-	-
No	16	184			
**Educational status**			0.758	4	0.944
None	0	4			
Primary/Arabic	1	6			
Secondary	3	36			
TTC/SON*	5	54			
Polytechnic/University	7	84			
**Marriage setting**			1.924	1	0.165
Monogamous	14	170			
Polygamous	2	8			
**Gravidity**			2.556	6	0.862
1	3	42			
2	2	25			
3	4	37			
4	5	51			
5	0	14			
6	1	11			
7	1	4			
**Booking status**			1.547	1	0.214
Booked	14	135			
Unbooked	2	49			

**Table 4:** prevalence of hepatitis B surface antigenaemia across occupations

Occupation	Maternal HBsAg		Occupation-based prevalence
	Positive Negative		
Unemployed	0	40	0.0
Trading	0	19	0.0
Civil servant	8	69	10.4
Hospital-related	6	27	18.2
Hairdressing	1	5	16.7
Others	1	24	4.0

Chi square 11.532, df = 5, p = 0.042

**Prevalence of hepatitis B surface antigenaemia across occupations:** the age-based prevalence (Table 5) was highest among teenage mothers (20%) and lowest among women older than 34 years (5.7%). Seven women (3.5%) had bodily inscriptions (scarification/tribal marks and tattoos) while 193 women (96.5%) did not have any form of bodily inscriptions. Five (5) out of the 7 women (71.4%) who had bodily inscriptions were hepatitis B surface antigen positive. Blood transfusion was not significantly associated with hepatitis B surface antigenaemia as none of the 16 women who tested positive for hepatitis B surface antigen had ever been transfused. Six (6) out of the 46 women (13%) who had undergone surgical/dental procedures tested positive for hepatitis B surface antigen, and this is not statistically significant (χ^2^=2.065, p=0.151, df=1). Similarly, none of the women who were hepatitis B surface antigen positive in this study had a past history of jaundice.

**Table 5:** prevalence of hepatitis B surface antigenaemia across age groups

Age group (yrs)	Maternal HBsAg		Age-based prevalence
	Positive	Negative	
≤19	1	4	20.0
20-24	1	10	9.1
25-29	7	64	9.9
30-34	5	73	6.4
≥35	2	33	5.7

Chi square 1.846, df = 4, p = 0.764

## Discussion

The sero-prevalence of hepatitis B surface antigenaemia from this study was 8.0%. This value places Ekiti State as an area of high endemicity for hepatitis B virus infection according to the classification by the World Health Organization [9-11]. The sero-prevalence value from this study is similar to the finding of Eke *et al*. where a value of 8.3% was reported for pregnant women at Nnewi in Nigeria [12] and the study by MacLean where a value of 8.0% was found among Malian pregnant women [13]. It is however higher than 2.19%, 2.5%, 4.4% and 3.5% found by other workers in Benin City, Port Harcourt, Tanzania, Lagos, and Bangladesh respectively [14-18]. A higher value (11%) was reported by Mbaawuga [19] among pregnant women in Makurdi and 18.5% was found among Brazillian pregnant women [20]. It is not surprising that the sero-prevalence value of this study is similar to that reported by Eke *et al*. considering the dynamics of the population studied which is similar to that of this present study in many respects. Studies that reported lower values from Benin City, Lagos and Port Harcourt could be as a result of the low-risk nature of the study population, better standards of living in those cosmopolitan towns, and higher sanitary conditions. On the other hand, investigators who determined the sero-prevalence for hepatitis B using all available markers for HBV in conjunction (such as HBsAg, anti-HB core, anti-HBs, envelope antigen and anti-envelope) tend to report higher values as in the case of Bertolini among Brazillian women [20].

The sero-prevalence of hepatitis B surface antigenaemia for this study was highest among pregnant women younger than 20 years of age with a value of 20.0% and lowest among women older than 34 years. This is in agreement with the finding at Nnewi12 where the highest reported prevalence was equally among younger women (20-24 age group), and the study by Dwivedi [21] among Indian women which showed the highest sero-prevalence for hepatitis B surface antigen among the 21-25 age group. Conversely, however, in another study by Bani among pregnant women in Saudi Arabia, the lowest prevalence was reported among women less than 20 years [22]. The higher prevalence noticed among younger women in this study could be a reflection of the changing trend in sexuality. Sexual debut is occurring at an earlier age and there is a possibility of a tendency towards high-risk sex during the teenage years [23,24]. Younger women tend to have unprotected intercourse, experiment with sex and are less likely to be able to negotiate safe sex [25]. Also, tattooing is a recent fad among the young which can also contribute to the higher prevalence among them considering the relatively higher infectiousness of hepatitis B virus when compared with the dreaded HIV [14]. In addition, this finding could also be a pointer to the impact of vertical transmission on adult prevalence of hepatitis B infection as these teenagers could represent chronic carriers that have been infected since childhood.

The neonatal sero-prevalence of hepatitis B surface antigenaemia for this study was 0.5 %. This value is in tandem with studies from Nigeria and those from other African countries with values less than 1%; Onakewhor reported 0.96% [14] among neonates in Benin-city and 0.93%was reported in Libya by El-Magrahe *et al*. [26]. Higher values were reported from other studies; neonatal sero-prevalence of 1.3% was reported for neonates in Bangladesh, 3.9% in Tanzania, and 6.7% in China [16,18,27]. The relatively low neonatal sero-prevalence value found in this study could suggest that the women in this study were of low infectivity status. While HBsAg is a good indicator of previous exposure to hepatitis B virus, it might not detect cases of early infections, hence it is not surprising that studies where hepatitis B viral DNA and anti-core antibody were determined on the neonates showed higher neonatal sero-prevalence values [18,27]. The vertical transmission rate for this study was 6.25%. This is lower than values reported in most other studies; Onakewhor´s study reported a value of 42.86% among neonates in Benin-city, Nigeria [14], 11.8% was reported among neonates in Tanzania [16], 12.78% was reported in Bangladesh [18], while 60.9% was found in a Libyan study [22]. On the other hand, vertical transmission of zero was reported in a study by Deveci *et al*. in Turkey [28]. Surprisingly in the Turkish study, even neonates of women who were envelope antigen-positive were not affected by the hepatitis B virus.

The low vertical transmission rate found in this study could be a pointer to the fact that the studied population is of low infectivity status. Furthermore, it could also be a reflection of the virulence of the hepatitis B virus strains in the locality. Maternal viral load, envelope antigen positivity and acute infection towards term are factors that determine the vertical transmission rate of hepatitis B infection [29,30]. The viral load of the women in this study was not known and it was also not known whether they were acutely infected in pregnancy or chronically infected. Vertical transmission is higher in women infected during pregnancy [31]. The study also considered risk factors for hepatitis B infection among pregnant women. Occupation and presence of bodily inscriptions (such as scarification, tribal marks, and tattoos) are the risk factors associated with hepatitis B surface antigen positivity. While campaigns against tribal/fascial markings are beginning to yield dividends as the practice of tribal marking seem to be declining, it is alarming that the practice of tattooing and other bodily inscriptions is on the increase and this trend can negate any gains from a reduction in the practice of tribal/fascial marking. Other risk factors such as previous history of blood transfusion, previous surgical/dental procedure, past history of jaundice, educational status, marriage setting and number of previous pregnancies were not statistically significant risk factors for hepatitis B infection in this study. This finding is consistent with the results of many studies [17,32,33].

Occupation in the health sector and hairdressing are predisposing occupations to hepatitis B infection as revealed by this study. Six (6) out of the 33 women (18.2%) whose occupation was health-related tested positive to surface antigen; these women were essentially health attendants and nurses. This finding is similar to Eke´s finding in Nnewi where 22.2% of the health workers in their study were positive to HBsAg [12]. In Eke´s study, it was also noted that health personnel in high-risk departments like blood transfusion services and theatres might be more at risk than such workers in pharmacies and outpatient departments. Workers in these departments are more in contact with body fluids and secretions and the practice of universal precaution is not well entrenched especially among the junior health workers such as cleaners and attendants. It has been said that the hepatitis B virus is over 100 time more infectious than the human immunodeficiency virus (HIV), requiring only 0.00004 ml of infected blood for its transmission compared to 0.1 ml for HIV [14], the viral particles can still be infective even on dried surfaces. One (1) out of the 6 hairdressers in this study tested positive for HBsAg (16.7%). This could be explained by the fact that they equally make use of needles and other sharp objects in the course of their daily business and this exposes them to similar risk as health workers. In addition, while it is expected of most health workers to be aware of and practice universal precautions, the same cannot be categorically said of hairdressers. This high sero-prevalence value among them is quite alarming and deserves urgent attention as they can form a major portal for disseminating hepatitis B infection among the female population who can then perpetuate the infection among their offspring. One of the key limitations of this study is its cross-sectional design, which restricts the ability to establish causality between maternal hepatitis B infection and vertical transmission to neonates. As the study only provides a snapshot of maternal and neonatal hepatitis B surface antigen (HBsAg) status at the time of delivery, it does not account for potential changes in maternal infection status during pregnancy, or variations in neonatal infection risk postnatally.

In addition, the sample size, though adequate for the study objectives, was limited to two hospitals in one State in Nigeria, which may reduce the generalizability of the findings to other regions or populations with different demographics or healthcare practices. Additionally, the reliance on the DiaSpot® rapid screening test, while convenient and widely used, may not be as sensitive or specific as more advanced diagnostic methods, potentially leading to false positives or negatives. Another limitation is the exclusion of certain groups, such as women with intrauterine fetal death or those with multiple pregnancies. This could introduce selection bias and limit the applicability of the findings to a broader population. Furthermore, the study did not investigate the long-term health outcomes of neonates who tested positive for HBsAg, limiting insights into the clinical significance of neonatal hepatitis B infection. Despite these limitations, the study has several strengths. It is one of the few studies conducted in this region of Nigeria, providing valuable data on hepatitis B prevalence and vertical transmission risks in a population where such information is scarce. By recruiting a diverse sample from two tertiary hospitals, the study captures a range of socioeconomic and clinical profiles, enhancing its relevance to the local population. The systematic collection of maternal and neonatal data, including risk factors for hepatitis B, allows for a comprehensive analysis of potential predictors of vertical transmission. Additionally, the use of a standardized rapid screening method ensures that the study procedures are easily replicable in similar settings, which is essential for informing public health interventions.

## Conclusion

This study has been able to prove that Ekiti State is an area of high endemicity for hepatitis B infection. It has also been shown in addition that despite this high endemicity, the neonatal sero-prevalence and vertical transmission rate of hepatitis B infection are relatively low. Furthermore, this study has revealed that occupation in the health sector, hairdressing, and the presence of bodily inscriptions are significant risk factors for hepatitis B surface antigen positivity. The findings of this study have also proven that though the risk factors for hepatitis B infection are well known, specific risk factors for each geographical location differ hence studies like this should be done to identify these location-specific risk factors and their determinants in order to effectively combat the disease. High endemicity of hepatitis B virus infection in pregnancy was demonstrated in the study. It has, therefore, become imperative to strengthen the structure of the various preventive arms of hepatitis B infection in pregnancy such as advocacy on increased community awareness and handling of risk factors, widespread vaccination campaigns, and routine testing during prenatal care.

### What is known about this topic

- Hepatitis B is a significant global public health issue, with approximately 2 billion people affected and 350 million chronic carriers worldwide;
- Hepatitis B can be transmitted from mother to child during pregnancy, with transmission rates varying depending on the trimester of infection;
- Nigeria has a high prevalence of Hepatitis B, with an estimated 18 million infected individuals.

### What this study adds

- This study provided specific data on the sero-prevalence of maternal and umbilical cord hepatitis B surface antigenaemia in a south-western Nigerian State, contributing to regional epidemiological data, which was previously lacking;
- The study identified that certain occupations, particularly those related to healthcare and hairdressing, are significant risk factors for hepatitis B surface antigenaemia among pregnant women;
- The study found a relatively low sero-prevalence of hepatitis B surface antigenaemia in neonates (0.5%) and a vertical transmission rate of 6.25%, suggesting that the infectivity status of the mothers in this population may be lower than previously assumed.
